# Where art thou? Reflecting on auditory hallucinosis

**DOI:** 10.1192/j.eurpsy.2023.1301

**Published:** 2023-07-19

**Authors:** I. Ganhao

**Affiliations:** Centro Hospitalar Psiquiatrico de Lisboa, Lisbon, Portugal

## Abstract

**Introduction:**

Hallucinosis has evolved out of classification systems but what about patients who present with exclusive or almost exclusive hallucinations? Auditory hallucinations are especially likely to swiftly be considered due to psychiatric illness.

An elderly patient with chronic auditory hallucinations without other significant psychopathology nor other symptoms prompted reflection and literature review.

**Objectives:**

To review differential diagnosis of auditory hallucinosis.

**Methods:**

Pubmed search for terms: auditory and hallucinosis.

**Results:**

Hallucinations should be evaluated according to: type, onset and evolution, association with physical and /or neurological symptoms, association with other hallucinations and/or other psychopathology, characteristics.

Auditory hallucinations may present along a continuum from tinnitus, simple, verbal, musical.

The Pubmed search retrieved articles pertaining to auditory hallucinations associated with:

1. Sensory deprivation; 2. Hearing loss, auditory Charles Bonnet syndrome; 3. Dementia, neurodegenerative disorders; 4. Brainstem lesions; 5. Other central nervous lesions: thalamus, temporal, other; 6. Epilepsy; 7. Tic disorders; 8. Alcohol use disorders; 9. Borderline personality disorder; 10. Others.

**Conclusions:**

Patients presenting with auditory hallucinosis should be carefully evaluated to exclude non-psychiatric disorders.

In some patients, such as the one who prompted the review, an identifiable cause may not yet be found.

**Disclosure of Interest:**

None Declared

